# Morphological Characterization of Indoor Airborne Particles in Seven Primary Schools

**DOI:** 10.3390/ijerph17093183

**Published:** 2020-05-03

**Authors:** Susana Pallarés, Eva Trinidad Gómez, África Martínez, Manuel Miguel Jordán

**Affiliations:** 1 Instituto de Salud Carlos III, C/Sinesio Delgado 4, 28029 Madrid, Spain; spallare@gmail.com; 2 Department of Agricultural and Environmental Sciences, Jaume I University, Campus Riu Sec s/n, 12071 Castellón, Spain; evatrinidadgomez@gmail.com; 3 Agricultural Economics Department, Cartographic Engineering, Graphic Expression in Engineering, Miguel Hernández University of Elche, Ctra. Beniel km 3,200, 03312 Orihuela (Alicante), Spain; africa.martinez@umh.es; 4 Department of Agrochemistry and Environment, Miguel Hernández University of Elche, Avd. Universidad s/n, 03202, Elche (Alicante), Spain

**Keywords:** indoor airborne fine particles, primary schools, mineral particles, spherical particles, carbonaceous particles, ceramic cluster

## Abstract

This paper focuses on a study of fine (less than 2.5 µm) airborne particles collected inside seven primary schools located on a coastal Mediterranean area which has a significant industrial hub for the processing of clays and other minerals as raw materials. Of the seven schools, three are located in an urban area 20 km away from the main industrial hub, three are in a town located next to the industrial estate, and one is in a rural location, 30 km from the industrial area. The objective of this study is to identify the main types of particles found in the three examined environments. The particle matter identified in the fine particle samples is grouped into three main groups: mineral compounds, particles from combustion processes and phases emitted in high-temperature industrial processes. The mineral particles, which can come from natural or anthropogenic emissions, have been classified depending on their morphology as isometric allotriomorphs or subidiomorphs, with a tabular habit; acicular forms; or pure crystalline forms. Compounds from combustion processes have two types of morphologies: spheroid and dendritic soot particles. Additionally, in smaller quantities, spherical particles associated with high-temperature industrial processes, such as the emissions of ceramic tile-firing and frit-melting processes, are identified. A summary table is shown, which lists the characteristics, as well as the most significant origin of the main particles identified in the fine (<2.5 μm) airborne particles collected inside primary schools located in three different environments (urban, industrial and rural). A visual scale has been established based on the number of particles observed in the samples of the atmospheric particulate fraction between the sizes of 2.5 and 10 µm collected inside the schools. The ratios Ca/Si, S/Si, S/Ca and (Si+Ca)/S have been established. Results obtained may be useful in epidemiological studies in the ceramic cluster area in order to estimate children’s exposure to different indoor primary school microenvironments. Effective policies and mitigation measurements for the protection of children’s health should be carried out in this highly industrialized area.

## 1. Introduction

Indoor particle concentration is the result of a number of factors, the most important of which are the indoor particle generation rate, the outdoor particle concentration, the air exchange rate, particle penetration efficiency from the outdoor to the indoor environment, and the particle deposition rate on indoor surfaces [1,2].

There can be several sources of particle matter inside a building: cleaning products, kitchens, heating systems, furniture or clothing, resuspension due to the movement of people, etc. However, in the absence of major sources of indoor particle emission, the registered concentrations are closely related to the outdoor particles, and in some cases, the concentration of contaminant particles inside is the same or greater than outside [3]. Atmospheric particles, similar to other types of atmospheric contaminants, are a result of both natural contributions as well as waste from human activity. Both depend, respectively, on the area’s climate and socio-industrial characteristics.

This study is focused on primary schools located on a stretch of Castellón province from the coastal basin known as Plana de Castellón, where the province capital is located, to a mountainous inland area. In the area of study, there are significant anthropogenic sources that produce particles including traffic, emissions from the key ceramic industrial cluster, and other emissions from different industrial sectors of lower importance in the province, such as the petrochemical complex of Castellón’s port area. The schools studied are located in the ceramic cluster of the province of Castellon (Spain) with an important zone dedicated to the treatment of ceramic raw materials. This ceramic cluster is a strategic area in the framework of European Union (EU) pollution control. Around 80% of the EU ceramic tile and ceramic frit factories are concentrated in two areas [4,5], forming two ceramic clusters: Castellón (Spain) and Modena (Italy). The manufacture of ceramics can produce the emission of gases from firing processes, which usually contain metals and other harmful substances. The diffuse emissions of CO_2_, fluorine, chlorine and sulfur from the ceramics industry in these European clusters suppose an important environmental and health risk [6,7]. The mineralogical analysis of settleable particles in the cluster ceramic of Castellon showed great homogeneity of the mineralogical composition of the samples [8]. The main mineral phases found were illite/muscovite (>40%) followed by quartz (>15%) and kaolinite (>10%). These minerals were associated with an anthropogenic origin in the area, as there are a number of companies engaged in the operation, crushing and fragmentation of clay. Furthermore, in the area of study, there is the concurrence of significant contributions of a natural origin, such as the climate characteristics of the coastal Mediterranean area, which negatively affect particle matter contents, increasing the levels of concentration due to three main natural processes: (1) resuspension phenomena; (2) material accumulation due to the low amount of rain and limited ventilation; and (3) the intrusion of contaminants from long-distance transport [9,10,11]. Scanning electron microscopy (SEM) has been used as a supplementary technique in order to complete the analysis of the samples collected inside the schools. Several authors have used this technique in particle characterization studies in order to establish origins and different morphologies [12,13,14,15,16,17,18,19,20,21,22,23].

The gravimetric and chemical composition of the particles makes it possible to learn their concentration levels, composition and shape. In order to determine the origin of the particles, X-ray diffraction (XRD) and fluorescence techniques (XRF) make possible the identification of the different types of minerals, glassy phases, combustion airbones, etc., which are present in the particle matter samples. However, in this study, the limited amount of sample collected in one filter does not allow us to use standard X-ray fluorescence (XRF) chemical analysis.

The main benefit of the SEM technique is that it can also be used with very small samples. It enables an individual analysis of the particles, whether isolated or as part of groupings, so that if there are morphological or chemical differences among them, they can be classified and, then, their origin can also be established [13].

SEM stands out as the best technique when conducting morphologic studies on air particles. Carbonaceous and soot particles are the compounds with most importance when studying the effects that particulate contaminants have on people’s health. These particles result from combustion, despite having, in general, a very homogeneous composition with mainly carbon contents, and can have very different morphologies depending on their origin (type of combustion, fuels, temperature, etc.), which makes SEM the best technique for studying them. For example, soot particles with a dendritic structure have the greatest tendency to interact with the nasal, thoracic and bronchial systems of human beings [12]. Furthermore, scanning electron microscopy allows learning of individual particle sizes. It also lets us establish ranges that the mentioned particles fit into.

The present work is the first effort to examine the morphology of indoor particles in primary schools in a European ceramic cluster. Results obtained may be useful in epidemiological studies. This study might contribute to the design of effective policies and mitigation measurements for the protection of children’s health in this highly industrialized area. The main objectives of this paper are (i) to carry out a morphological study of the particles gathered inside primary schools located in a coastal Mediterranean area; (ii) to establish differences in size, concentration and composition of the atmospheric particles among seven schools sited in three different environments; and (iii) to classify the particles, know their possible origin and provide data for future epidemiological studies.

## 2. Material and Methods

### 2.1. Description of the Sampling Site

**Figure 1:**
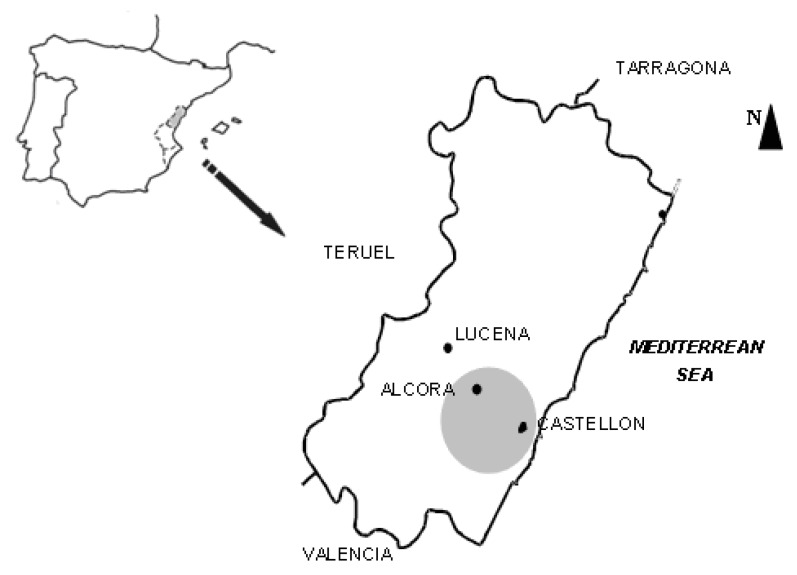
Location map of three different environments: urban (Castellón), industrial (Alcora) and rural (Lucena).

This study focuses on a stretch of the ceramic Spanish cluster that goes from Castellón city, located in the plane of Castellón, without natural barriers, passes through the town of Alcora located on the slope of a hill, and finishes in Lucena, located in the mountainous inland area (Figure 1). Three different environments (urban, industrial and rural) are represented by Castellón, Alcora and Lucena respectively. 

Three schools (S) were selected in the city of Castellón: S1, S2 and S3. All of them are located near a high density of traffic road networks. The selected three schools are located in different areas of the Castellón city, with different orientations (air currents and urban morphologies).

A key feature shared by the three schools in the town of Alcora is their proximity to an important ceramic industrial hub. Three primary schools were chosen—S4, S5 and S6—in different points of the town with different orientations and environments, with the same objective: to analyze the influence of these characteristics of indoor particle shapes and concentration levels.

In the village of Lucena, an area with a low concentration of industry and a low density of traffic, only one the school was selected (S7). Table 1 summarizes the characteristics of the selected primary schools. 

It is important to indicate that, for each school, classrooms with different volumes (m^3^) have been selected. The number of students for classroom is also variable. Typically, the number of children per classroom ranges between 25 and 40. However, in rural schools, this number is halved. In the case of S6, a classroom with a high number of students (60) was selected.

### 2.2. Sample Collection

The sampling period was for two years (July and August excluded for the holiday period). Samplings were performed for five to eight hours depending on the school activities. A monthly sample for each school was obtained.

The device RespiCon TM (Helmut Hund GmbH, Wetzlar, Germany) was used to collect the indoor airborne samples. This sampler is a multistage virtual impactor that simultaneously collects the ISO/CEN/ACGIH size fractions of inhalable, thoracic, and breathable particulate matter [13]. This collector traps airborne particles on three individual collection filters. At a flow rate of 3.11 L/min., the first stage separates out and collects the particles that are smaller than 2.5 μm. The second stage collects particles between 2.5 and 10 μm, while the third stage collects the remaining particles.

In order to collect indoor particles Quartz fibre and PTFE filters (37-mm diameter) were used. PTFE filters were only used in the first and second stages of Respicon TM. The filters were examined using SEM to establish physical particle size distribution. 

### 2.3. SEM Analysis

PTFE filters (1 cm^2^ approximately) were fixed onto an aluminum SEM stub using a conducting carbon. Table filters were coated with at least 30 nm of Au/Pd in order to get a higher quality secondary electron image.

Collected particles were analyzed using a scanning electron microscope (SEM) type LEO model 440. A micro-analysis equipment OXFORD EDX (Oxford Instruments, Abingdon, Oxfordshire, UK) was used to study these particles in detail. The geometric shapes observed revealed a possible anthropogenic source. The morphology and size of selected particles were analyzed using secondary electrons. Micrographs were taken at magnifications of 7500×. All measurements were done with a beam current of 10 nA at an accelerating voltage of 20.00 kV.

Taking into account the objective of valuing the importance of the different compounds (mainly mineral phases and particles from combustion processes) in the fraction of particles in suspension, we decided to carry out a comparative study by means of elemental distribution maps (mapping). This study involved analyzing and comparing particles with a high content of Ca, Si and S in the coarse fraction (2.5–10 μm) of suspended particles. For these chemical analyses, back-scattered electrons and the OXFORD EDX equipment were used, with the following working conditions: acceleration voltage 20 kV, energy range 0–10 keV, acquisition time 500s and 1000× magnification. The ZAF method for semi-quantitative analysis was used.

## 3. Results and Discussion

### 3.1. Identified Particles

Emissions produced outdoors (i.e., traffic, construction or industry emissions) penetrate the interior of buildings, and these represent the primary contribution to indoor air quality [19,22,23]. The use of natural ventilation favors, in general, indoor particle reduction. However, there are cases in which the orientation of the school encourages the penetration of particles aided by the wind. In these cases, it is advisable to ventilate certain times when the sea breezes and prevailing winds do not favor the entry of particles. Thus, many activities take place in the playgrounds located next to the classrooms. However, the emissions of heating systems, air fresheners, and cleaning products can produce other particles. 

The particulate matter identified by SEM in samples of this study were grouped into two main groups: (i) mineral particles and (ii) other phases originated by combustion processes or by processes at high temperatures.

#### 3.1.1. Mineral Particles

**Figure 2:**
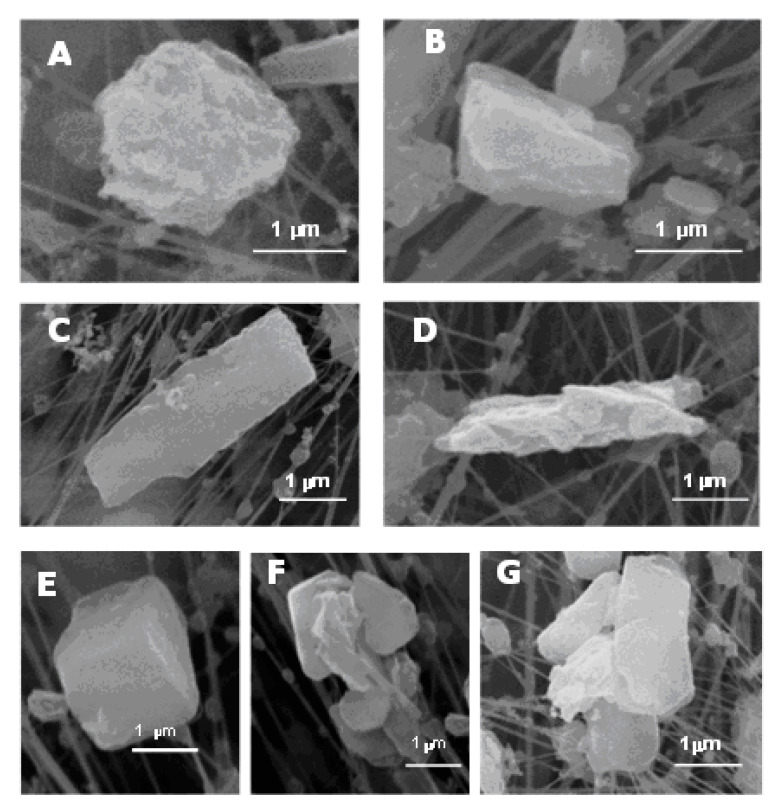
Scanning electron microscopy (SEM) images of suspended particulate matter: (**A**) allotriomorphous particle; (**B**) subidiomorphous particle; (**C**) tabular; (**D**) acicular; (**E**) cubic crystal; (**F**) rosette-shape particle; (**G**) mineral aggregates.

The mineral phases found in filters taken inside the schools have been classified according to their morphologies (Figure 2).

A. Isometric allotriomorphs. These are particles without predominant growth in any direction and without faces with external crystalline appearance, without defined ridges (allotriomorphic, Figure 2A), or with some sign of crystallization on their exterior (subidiomorphic, Figure 2B).

In general, the allotriomorphic particles are silicates or carbonates. On some occasions, these phases have rounded borders that indicate signs of degradation.

The origin of the subidiomorphic particles, including their chemical and mineralogical composition (carbonates and silicates) is associated with industrial processes used in the ceramics industry (milling, crushing, etc.). These particles show defined edges.

B. Tabular habit (Figure 2C). The composition of these types of particles is mainly aluminum silicates which were identified by [16]. These particles are related to natural emissions or clay treatments. Sometimes, they form agglomerates with chlorates and/or sulphates as bonding materials. 

C. Acicular morphology (Figure 2D). These particles are crystals with a very elongated habit. Their chemical composition is variable. Previous studies [16] found the presence of calcium, sulfur, and potassium or sodium.

D. Pure crystalline forms. These particles are called “neoformation particles” because, in a lot of cases, they crystallize in the atmosphere after the emission. Halite and gypsum phases have been identified in some samples. Halite (Figure 2E) in atmospheric samples is a component of marine aerosols [24,25]. The neoformation of secondary gypsum (Figure 2F) is produced during the transportation of pollutants, which come from the oxidation of sulphates and their precipitation or conversion to carbonates (CaCO_3_). The main sources of the sulfur oxides (SO_x_) in the area of Castellón are the BP petrochemical complex [16] and the traffic. In the studied ceramic cluster, natural gas (with low content in S) is usually used in the industry [16].

E. Mineral aggregates (Figure 2G). The origin of these aggregates is the different mixtures of raw materials usually used in the sector of industrial ceramics.

#### 3.1.2. Particles Emitted During Combustion Processes

**Figure 3:**
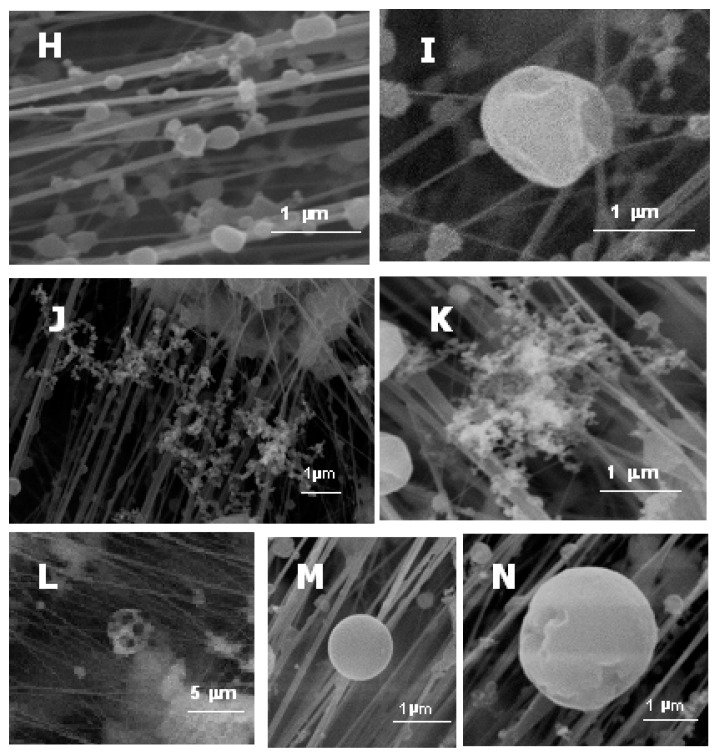
SEM images of suspended particulate matter: (**H**,**I**) sphere-like particles, (**J**,**K**) soot dendritic aggregates, (**L**) fuel oil particle, (**M**,**N**) spherical particles.

This group is made up of particles with variable morphologies, but which have a high content of carbon. This is the only feature they have in common. The main carbonaceous particles identified in this research were sphere-like particles (Figure 3H,I) and dendritic soot aggregates (Figure 3J,K). At a lower percentage, particles with rounded morphologies were also found (Figure 3L).

A. Sphere-like particles or rounded forms. These groups include rounded morphology particles formed by a carbon matrix containing trace elements (Na, K, Fe, S, etc.) included in graphitic phases. These spherical or spheroid particles originate from carbonized particles derived from incomplete oxidation processes from different fuel sources (carbon, gasoline, etc.) or pyrolysis [12,26].

B. Dendritic soot aggregates. Organic compounds, after combustion, condense in the air to form spheres (<100 nm) that bond to create dendritic chains of soot, which contains a lot of nanocomponents [20]. In a previous study in the ceramic cluster, soot aggregates were formed by Si, S, K and Fe and high contents of C [27].

C. Particles from fuel oil combustion. These particles are characterized by having a rounded shape and being porous. These particles are usually hollow due to the ejection of inner material and to the mechanical stability of the surface, which allows the formation of this type of particle [14].

#### 3.1.3. Particles Originated by High-Temperature Industrial Processes

These particles present spherical morphology (Figure 3M,N), a geometrical shape which excludes any crystalline genesis process. Their origin is associated with frit-melting processes. Most of them have plain texture on the surface with small cracks [28].

#### 3.1.4. Other Particles

In this section, small fibers that have been found, sporadically, in some samples in one school are grouped. These fibers can have properties, such as aerodynamic forms, which are not present in compact particles [29]. The fibers can be both organic and inorganic in nature. The EDX analysis of one of these fibers shows that the main element is carbon. Their composition and their morphology allow us to link these fibers to compounds of an organic nature.

### 3.2. Fine Fraction (<2.5 μm) Characterization

Table 2 summarizes the results obtained in the morphological analysis of the samples of the fine fraction of particles within schools.

The main difference between urban and industrial locations is that the former contain a greater number of particles which are smaller in size. High levels in most industrial locations are due to the sum of particles from traffic and minerals, which are found in smaller amounts but have greater weights.

Of the three schools in the urban environment, the school S2 has higher concentrations of mineral particles (in quantity and size) but the lowest levels of particles, which are smaller than 2.5 µm in this type of location. This observation is associated with the presence of a non-paved esplanade next to the sampling point, which is used for parking, and with the non-existence of high-density traffic routes.

Schools located in urban locations have higher concentrations of particulate matter from combustion processes than the ones found in industrial or rural environments. This fact is due to the greater proximity to the sample point of routes with high traffic density. Furthermore, a higher proportion of sphere-like particles compared to dendritic aggregates were found in urban locations.

In the area of study, both spheroids and dendritic soot aggregates essentially come from the combustion processes of motor vehicles, as the ceramic industry mainly uses natural gas, which is a particle free source (source: www.mityc.es). The difference in content and distribution among compact carbon, spheroid and dendritic soot aggregate masses is due to the proximity of roads [26] (the number of particles decreases by 50% when located 100 m from a road), the speed of vehicles on those nearby roads and the transport capability of the various types of morphologies, among others reasons. Soot aggregates can travel large distances through the air [19] due to their low density and small size.

According to Kittelson [30], when the speed of the traffic increases, there is a high concentration of the number of particles, but they have smaller size and volume, which means that the final mass concentration decreases at higher speeds.

As the speed decreases, there is greater emission and larger particle sizes and volumes. The optimal level of a lower mass concentration of particles occurs at speeds between 90 and 100 km/h. The speed of motor vehicles also has a certain amount of influence on the morphology of the particles that come from combustion. The higher the speed, the greater the possibilities of nucleation taking place, and, therefore, the greater the possibilities of producing soot. For example, some authors [31] showed that the nucleation processes that lead to the creation of soot follow this trend: they do not appear at speeds of 50 km/h, as their creation begins at speeds of around 100 km/h, and they become stable from 120 km/h.

From these observations, one can surmise that the reason why there are greater numbers of spheroid-type particles as a result of combustion processes in urban locations is because there is more traffic travelling at slower speeds (traffic jams, traffic lights, etc.). An increase in the total number of particles in urban locations is linked to an increase of particles from combustion processes (mainly spheroid particles).

Locations in an industrial environment have the greatest concentration of mineral particles. This is because the industry in the area is based on processing mineral raw materials. The increases registered in the total number of particles in locations S5 and S6 are linked to an increase in this type of particle. Prior studies conducted in this town established that the enrichment of these particles is within the 2 to 4 µm range.

Of the three schools located in an industrial environment, school S5 had the greatest concentration of particles. It is located in an open area where it directly receives the morning sea breeze, which carries the contaminants from the industrial area located west of the town.

The increase in the total number of particles is mainly due to the increase in mineral particles (as has been mentioned) and also due to the fibers that were only found in this location.

Greater amounts of dendritic soot aggregates are found in locations where the direct source of traffic is located further away, as this type of particle can travel long distances in the air [20].

The sizes of the dendritic soot aggregates increase as we get further away from major sources of traffic, due to the formation of aggregates. After combustion, organic compounds are condensed in the air, which create very small (less than 100 nm) spheres, which then form aggregates in the atmosphere and create dendritic soot chains [20]. Therefore, increasing the transport time will enhance the size of soot aggregates, which is why larger chains are found in rural locations compared to those in industrial sites, whereas the latter are larger than those found in sample points located in urban environments.

The coagulation that takes place in the creation of soot (just like surface growth and oxidation) can affect the size and density of the particle. Soot is formed in a very heterogeneous process in the combustion chamber.

The influence of the type of fuel is essential in this process, with diesel engines that have a much higher particle emission factor compared to injection and petrol engines (30–50 mg·km^−1^ diesel, 6–13 mg·km^−1^ injection and 0.5–2.5 mg·km^−1^ petrol) [30]. Furthermore, the difference in fuel can also be seen in the size of the particles, with average diesel engine particle sizes ranging from 60 to 120 nm, and from 40 to 80 nm for petrol engines [19,30]. This paper will not assess the influence of the type of fuel because the data on the number of vehicles is not available. Lastly, particles from natural gas emissions are smaller than those from diesel and petrol, and range between 10 and 70 nm [19].

Neoformation compounds (halite and gypsum cubes) are found in places that are located in industrial and rural environments. Although the urban location is closer to the source of origin, these compounds are created afterwards in the atmosphere while being carried to areas that are further away from the source of emission. They are generally found in larger numbers in hot seasons because the ions that trace marine influence, as happens with nitrates and sulphates, have greater spreading (transport) capabilities through fluvial channels during these seasons [27].

### 3.3. Coarse Fraction (2.5–10 μm) Characterization

A visual scale has been established based on the number of particles observed in the samples of the atmospheric particulate fraction between the sizes of 2.5 and 10 µm collected inside the schools. This way, low concentration has been linked to the lowest number of particles corresponding to the month of March for location S7 (rural). The highest concentration was registered in the month of February in location S5 (industrial).

It can be seen in Table 3 how the largest concentration of particles was registered in the school located in an industrial location, S5, with high levels also being registered in location S6. School S4, despite being industrial, does not follow the same trend and has noticeably lower particle figures. Regarding the number of particles collected in the three urban locations (S1, S2 and S3), it is worth noting that it was similar (generally medium-high), although a slight increase was observed for location S3. School S7 (rural location) is where the lowest figures of particles in the PM2.5–10 range were observed, as it happens with particles smaller than 2.5 µm. Moreover, based on the elemental distribution maps obtained, the average percentage for the analyzed elements (Si, Ca and S) has been analyzed in the different locations using the computer program SigmaScan Pro 5, which is an image processer. The percentage of the total sample area occupied by the different elements has been calculated in proportion to their amounts for comparison. Furthermore, the values of Si and Ca have been added together to calculate the mineral contribution, and different ratios have also been established (Ca/Si, S/Si, S/Ca and (Si+Ca)/S). The results obtained can be seen on Table 4.

Firstly, in reference to the contents in percentage obtained for elements Si, S and Ca, it can be stated that:

(a) School S2 is the urban location with the lowest percentage for all elements, whereas school S1 has the highest percentages of S.
(b) School S7 (rural) has the lowest values of Ca, Si and S of all the studied schools.
(c) Industrial locations S5 and S6 have similar values of Ca and Si; however, the percentage of S is greater in school S6. Location S4 has the lowest values of all industrial schools.

Secondly, when taking into account the calculated ratios:

(d) The highest proportion of sulfur compounds was registered in school S1 (urban); this fact can be observed in the results obtained when calculating ratios S/Si and S/Ca, both greater than 1.
(e) Very similar values were obtained in school S6 for the S/Ca and S/Si ratios, which gives us similar values for particles rich in Si and Ca. This same situation occurs in school S5, where a value of 0.79 was obtained for the ratios S/Si and S/Ca, which is also the lowest value obtained. This fact tells us that, as well as having similar values for phases with Si and Ca contents, S5 is the school with the highest amount of mineral compounds regarding phases resulting from traffic.

Industrial schools have higher concentrations of particulate in the 2.5 to 10 µm range than urban schools. This is because this size range is enriched by the most common mineral compounds in industrial schools close to emission sources of the ceramic sector. The ceramic industry’s activity generates the emission of quartz particles, clay minerals (illite and kaolinite), Na-Ca plagioclase, feldspars and carbonates (kaolinite and dolomite), which come from moving mineral raw materials whose size ranges between 2 and 100 µm [32]. The high contents of Si and Ca detected in both schools confirm this fact.

School S4 presents an abnormal behavior compared to the other industrial locations. In this school, noticeably lower average concentrations of particulate matter between 2.5 and 10 µm were detected, and the elements analyzed by mapping (Ca, Si and S) are the lowest of all industrial schools. The factor that has the greatest impact on these differences is the ventilation which, along with the orientation and structure of the building, divided by classrooms with windows that lead out to an inner courtyard surrounded by the adjacent buildings (a closed area with a shielding effect that blocks the entry of contaminants), leads to a decrease in levels of particulate matter. This school, unlike the other points studied, is constantly exchanging air with the outside, as one of the access doors to the terrace (the location where the equipment was located) is always open. Due to this constant circulation of air, no particle accumulation process takes place inside of the school, as happens in the other ones studied. Furthermore, it is located in a closed area that does not favor the contribution of particulate matter from the exterior. The effect of these features is that the concentrations obtained are less than expected.

Urban school S1 has the highest percentages of S of all the schools studied, a fact which is linked to the major influence that traffic has on this location. The main anthropogenic sources of S are SO_2_ and H_2_SO_4_, which come from the combustion of fossil fuels, which have traces of sulfur. Furthermore, SO_2_ is one of the main contaminants emitted by the petrochemical complex and the thermal power plant located west of this location, which has a greater influence on this sampling point than on any other school chosen in this town.

Winds have a great influence on the dispersion and transport of air pollutants. In the sampling area, the most important air currents are periodic land and sea breeze [5,16]. The directions and speed of these two wind components in the area vary depending on the topography. The change from one component to another depends on the hours of the day, and, therefore, the beginning and end of each breeze component depend on seasonality [3,5]. One of the limitations of this work has been not being able to link the morphology of the captured particles with the breeze regime, since the sampling is carried out continuously, collecting one filter per month.

The smallest percentages of all elements were registered in rural school S7. However, figures for the ratios of S/Si and S/Ca have similar values to those obtained in the urban locations and are greater than those in industrial locations. The cause of this observation is that, as mentioned earlier, the main source of S is the combustion of fossil fuels. These processes emit low-density particles (with S), have high transport capabilities and can travel long distances from their emission source [11,16].

## 4. Conclusions

Mineral particles which can come from natural or anthropogenic emissions were classified depending on their morphology: as isometric allotriomorphs, with a tabular habit; crystalline; or acicular forms. Two types of morphologies (spheroid and dendritic soot particles) have been found in particles from combustion processes. In lesser amounts, spherical particles associated with high-temperature industrial processes, were identified. This research lists the characteristics (morphology, type and sizes), as well as the most significant origins of the major particles identified in the fine (less than 2.5 μm) airborne particles collected within schools located in three different environments.

A visual scale has been established based on the number of particles observed in the samples of the atmospheric particulate fraction between the sizes of 2.5 and 10 µm, which were collected inside the schools. Values of Si and Ca obtained have been added together to calculate the mineral contribution. The ratios Ca/Si, S/Si, S/Ca and (Si+Ca)/S have also been calculated.

Results obtained may be useful in epidemiological studies in the ceramic cluster area, in order to estimate children’s exposure to different indoor primary school microenvironments. Effective policies and mitigation measures for the protection of children’s health should be carried out in this highly industrialized area.

## Figures and Tables

**Table 1:** Main characteristics of the studied schools. Legend: N: North; S: South; E: East; W: West.

School	Site	City Zone	Traffic Density	Classroom Volume (m^3^)	Orientation of Windows	Number of Students
S1	Urban	E	High	268.03	WNW	40
S2	Urban	NW	Medium	159.56	SSE	21
S3	Urban	W	High	173.49	ESE	20
S4	Industrial	SE	Medium	136.17	WNW	25–30
S5	Industrial	E	Medium	109.09	ENE	26
S6	Industrial	SW	Low-Medium	197.67	SSE	60
S7	Rural	SE	Low	182.60	SE	12–8

**Table 2:** Results of fine fraction (<2.5 μm) characterization.

Characterization		Type of Location Environment		
		Urban	Industrial	Rural
**Amount**	Total particles	High	High	Medium
	Mineral phases	Medium	High	Medium
	Combustion compounds	High Predominantly sphere-like particles	Medium Predominantly Dendritic aggregates	Low-Medium
**Size**	Mineral phases	(1–1.5) μm	(1–1.5) μm	(1–2) μm
	Sphere-like particles	(0.2–0.5 μm)	(0.2–0.5) μm	(0.2–0.5) μm
	Soot dendritic aggregates	<1 μm	until 2 μm	until 2.5 μm
**Spheres**		In all schools Diameter (0.6–0.8) μm	In S4 and S6 Diameter (0.8–2) μm	Not observed
**Fibres**		Not observed	Only in S5	Not observed
**Secondary particulate matter**			Cubic shapes and gypsum crystals (S5)	Cubic shapes (S7)

**Table 3:** Amount of coarse particles.

	Amount of Particles				
Title	Feb.	Mar.	May	Oct.	Nov.
S1	+		•		+
S2		+	•		++
S3	++		+	+	
S4		-	•		•
S5	+++		+++	+++	
S6		++	++	++	
S7		--	--	•	

S (school), (+++) very high, (++) high, (+) medium-high, (•) medium, (-) low-medium, (--) low, close to mean (blank).

**Table 4:** Percentages of the different elements analyzed by mapping and quotients calculated for each school.

	Schools						
	S1	S2	S3	S4	S5	S6	S7
Si	3.74	2.56	3.53	3.11	3.96	3.91	2.24
S	3.95	2.19	2.60	2.58	3.12	3.61	2.11
Ca	3.77	2.19	2.72	2.32	3.76	3.80	1.99
Si+Ca	7.51	4.75	6.24	5.43	7.72	7.71	4.23
Ca/Si	0.97	0.88	0.77	0.79	1.01	0.95	0.87
S/Si	1.08	0.88	0.78	0.86	0.79	0.93	0.92
S/Ca	1.18	1.01	1.01	1.11	0.79	0.97	1.07
(Si+Ca)/S	1.90	2.17	2.40	2.11	2.48	2.13	2.00
